# Arginine Exposure Decreases Acidogenesis in Long-Term Oral Biofilm Microcosms

**DOI:** 10.1128/mSphere.00295-17

**Published:** 2017-08-23

**Authors:** Ruth G. Ledder, Hitesh Mistry, Prem K. Sreenivasan, Gavin Humphreys, Andrew J. McBain

**Affiliations:** a Division of Pharmacy and Optometry, School of Health Sciences, Faculty of Biology, Medicine and Health, The University of Manchester, Manchester, United Kingdom; b Colgate-Palmolive Company, Piscataway, New Jersey, USA; University of Nebraska Medical Center

**Keywords:** arginine, dental biofilm microcosm, dental plaque

## Abstract

Arginine is an important pH-elevating agent in the oral cavity. It has been incorporated in oral hygiene formulations to mitigate sensitivity and to prevent caries. In this investigation, the effects of sustained arginine dosing of dental plaque microcosms on bacteriological composition and pH were evaluated under controlled conditions. Plaque microcosms were established in constant-depth film fermentors (CDFFs) using salivary inocula and fed continuously with artificial saliva. To simulate resting and cariogenic states, the CDFFs were supplemented with sterile water or 5% sucrose, respectively. Plaques were then dosed twice daily with a dentifrice with 1.5% arginine arginine added (DA) or without arginine (DN). This regimen continued for over 3 weeks, after which fermentors were maintained without dosing. Microcosms were analyzed by differential viable counting, with a pH microelectrode, and by eubacterial DNA profiling. Sucrose dosing was associated with significantly (*P* < 0.001) decreased pH, significantly (*P* < 0.05) increased counts of total aerobes, Gram-negative anaerobes, aciduric species, acidogenic species, arginine utilizing species, bifidobacteria, lactobacilli and streptococci, and significant (*P* < 0.05) changes in DNA profiles. Plaques dosed with DA had a significantly (*P* < 0.001) higher pH than those dosed with DN, with or without sucrose supplementation. Dosing with DA but not DN significantly (*P* < 0.05) decreased counts of all functional bacterial groups apart from the total anaerobes in cariogenic plaques, and in resting plaques, dosing with DA significantly (*P* < 0.05) decreased counts of streptococci, lactobacilli, bifidobacteria, and acidogenic bacteria. In summary, sustained exposure of oral microcosms to arginine in formulation significantly increased plaque pH and significantly reduced the viability of cariogenic bacterial species.

**IMPORTANCE** Arginine is used in dental health formulations to help prevent dental cavities. This study assessed the effects of the long-term dosing of laboratory dental plaques with an arginine dentifrice. Data indicate that the addition of arginine dentifrice during sucrose challenge significantly increased plaque pH, thus potentially mitigating cariogenesis. Counts of several functional groups of bacteria associated with tooth decay were significantly decreased in the laboratory plaques during exposure to the arginine dentifrice.

## Abstract (precis)

Arginine is used in dental health formulations to help prevent dental cavities. This study assessed the effects of the long-term dosing of laboratory dental plaques with an arginine dentifrice. Data indicate that the addition of arginine dentifrice during sucrose challenge significantly increased plaque pH, thus potentially mitigating cariogenesis. Several functional groups of bacteria associated with tooth decay were significantly decreased in the laboratory plaques during exposure to the arginine dentifrice.

## INTRODUCTION

Dental diseases affecting the hard and soft tissues represent some of the commonest diseases globally (1–3). Examples of these conditions include dentinal hypersensitivity, inflammatory conditions such as gingivitis, and carious lesions on the dentition. Thus, approaches to control these conditions are of considerable public health significance and normally involve brushing with dentifrice, the effectiveness of which can be augmented by the inclusion of ingredients such as fluoride and antimicrobial agents (4). The use of arginine, a basic amino acid, represents a distinctive strategy in the management of dental caries and dentinal hypersensitivity (5, 6). Arginine, when applied in combination with bicarbonate and calcium carbonate at alkaline pH, forms a protective layer of salivary glycoprotein (7), which is resistant to acid and fluid flow. This phenomenon is utilized for dentinal tubule occlusion, and the effectiveness of arginine formulations in relieving hypersensitivity has been demonstrated clinically (8–11). Protection may be conferred following a single use (12, 13) and following extended, repeated use (8), with effects noted for up to 8 weeks (14, 15). Additionally, longer-term (up to 2 years) clinical studies have evaluated the potential anticaries effects of arginine-containing dentifrice. These studies were conducted in a large population (up to 6,000 patients) and concluded that within 3 months of regular use, arginine-containing dentifrice significantly inhibited caries in comparison to a fluoride paste (16–18). Acevedo et al. evaluated the anticaries effects of both a sugarless mint (19) and a dentifrice (20) containing arginine bicarbonate. Both studies demonstrated a statistically significant inhibition of the onset of caries and its progression over 1 year and 2 years, respectively, which was attributed to the generation of ammonia via the metabolism of arginine by the oral microbiota.

Free arginine is present at micromolar concentrations in saliva (21) and is also liberated during the degradation of salivary proteins or peptides (22). In particular, histidine-rich proteins for example, may contain up to 20 mol% arginine (23). Pathways for arginine degradation by oral bacteria include the arginine deiminase system (ADS) that rapidly converts the available plaque arginine to ammonia (24). Hydrolysis of arginine to ammonia via the ADS has been shown to offer protection to less aciduric bacteria from plaque acidification (25–27) as well as synthesizing ATP for bacterial use (23). The ADS system occurs in a variety of the oral bacteria, including *Streptococcus gordonii* (28), *Streptococcus sanguinis* (29), *Streptococcus parasanguinis*, and some lactobacilli (23). Interestingly, strains of these bacteria have been previously associated with oral health. Socransky et al. (30), for example, correlated the presence of *S. gordonii* and *S. sanguis* with a lower incidence of bleeding on probing. Additionally, Jakubovics et al. (31) demonstrated that the presence of arginine was critical for biofilm formation in *S. gordonii*. Nascimento et al. (32) demonstrated that the increased availability of arginine in the oral environment through an exogenous source enhanced the ADS activity levels in saliva and dental plaque.

Supplementation of oral health care products with arginine has been clinically demonstrated to reduce dentinal hypersensitivity (9, 11, 33) and the onset and progression of caries (19, 20), and the routes of arginine metabolism by the oral microbiota have been documented (23). Less information is available, however, concerning the bacteriological effects of prolonged arginine exposure. *In vitro* studies of the effects of arginine on coaggregation and biofilm formation have suggested that arginine potentiates the effects of other antimicrobial agents as well as moderating plaque development (34). Koopman et al. (35) demonstrated that arginine-containing toothpaste affects the arginolytic capacity of saliva and reduces its sucrose metabolic activity.

Advances in *in vitro* modeling and analytical methods for monitoring bacterial communities mean that it is possible to resolve ecological perturbations due to arginine dosing. The present study therefore evaluated the effects of prolonged arginine within a dentifrice exposure of *in vitro*, saliva-derived dental plaque with and without sucrose dosing. Populations of oral bacteria were monitored using extensive differential culture targeted toward major functional groups of oral bacteria, including those capable of arginine metabolism and aciduric and acidogenic species. Plaque pH was monitored throughout, and to determine the effect of arginine on bacterial community composition, eubacterial PCR combined with denaturing gradient gel electrophoresis (DGGE) was used to objectively compare community compositions over time and during dosing.

## RESULTS AND DISCUSSION

It has previously been established that oral health care products containing arginine can lower the incidence of caries (19, 20) and reduce dentinal hypersensitivity (9, 11, 33). The present study utilized a range of variably selective agars for differential viable counting and eubacterium-specific DNA profiling to analyze plaques grown under various arginine dosing regimens in a previously validated continuous culture model. The data thus generated can be used to better understand the effects of prolonged exposure to arginine on bacterial composition and pH in dental plaques.

*In vitro* model systems have been previously used to evaluate the effects of various interventions, including triclosan (36–38), chlorhexidine (39–41), and enzymes (42), on dental plaques *in vitro* and to model caries-like lesions (43). These approaches have provided useful insights into the mode of action and bacteriological effects of a variety of antiplaque agents within steady-state *in vitro* plaque systems (40, 44, 45). In the present investigation, salivary microcosms were established within constant-depth film fermentors (CDFFs) under controlled environmental conditions and consistent nutrient availability to better determine the effects of arginine supplementation. CDFFs were run concurrently to reduce variation between runs (46). Microbial community dynamics were monitored by differential bacterial enumeration and eubacterium-specific PCR-DGGE. Additionally, localized effects upon plaque acidogenesis were determined using a pH microelectrode.

### Effects of arginine on functional groups of bacteria.

**FIG 1:**
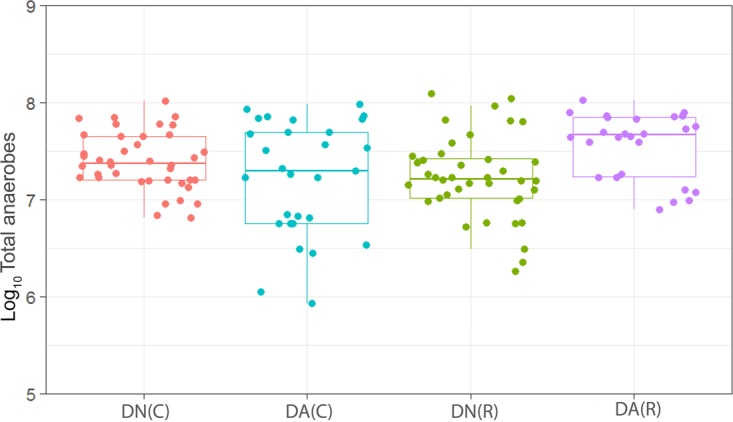
Distribution of the viable counts of total anaerobes in plaques dosed with (DA) and without (DN) arginine dentifrice in the presence (C, cariogenic) and absence (R, resting) of sucrose. Box plots: red, DN with sucrose; cyan, DA with sucrose; green, DN without sucrose; magenta, DA without sucrose. The horizontal bars within the boxes represent median values; the error bars indicate standard deviations. Each box plot represents the analysis of between 25 and 30 individual plaques. Dentifrice (DA or DN) and sucrose (5% [wt/vol]) or water were added to the fermentors every 6 h over a period of 22 to 29 days. Counts of total anaerobes were not significantly changed (*P* > 0.05) based on sucrose addition in plaques dosed with DN or based on DA addition in cariogenic plaques. Counts of total anaerobes were significantly (*P* = 0.035) higher in resting plaques dosed with DA than in those dosed with DN.

**FIG 2:**
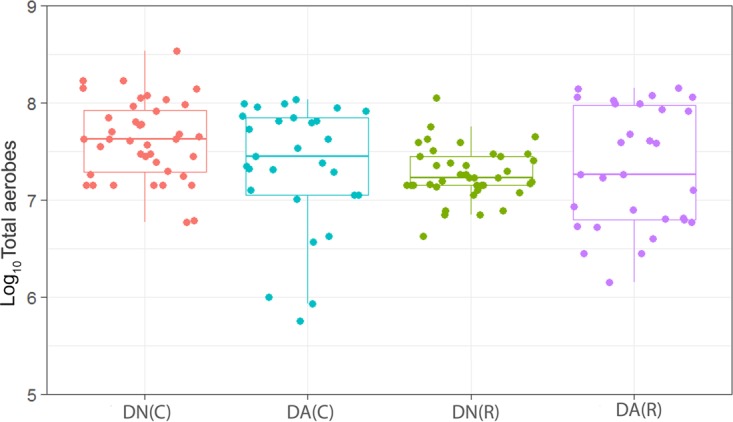
Distribution of the viable counts of total aerobes in plaques dosed with (DA) and without (DN) arginine dentifrice in the presence (C) and absence (R) of sucrose. Box plots: red, DN with sucrose; cyan, DA with sucrose; green; DN without sucrose; magenta, DA without sucrose. For details, see the legend to Fig. 1. Counts of total aerobes were significantly (*P* < 0.001) higher in cariogenic plaques dosed with DN than in resting plaques dosed with DN. Cariogenic plaque counts of total aerobes were significantly (*P* = 0.017) higher in DN-dosed plaques than in DA-dosed plaques. There was no significant difference (*P* = 0.60) between resting plaques dosed with DN and those dosed with DA.

**FIG 3:**
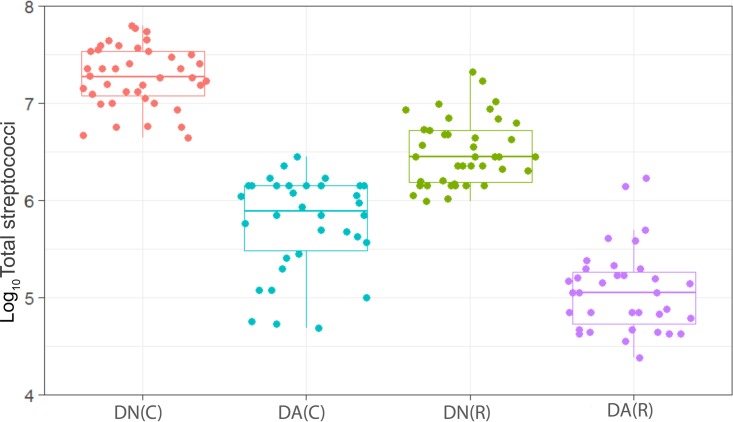
Distribution of the viable counts of streptococci in plaques dosed with (DA) and without (DN) arginine dentifrice in the presence (C) and absence (R) of sucrose. Box plots: red, DN with sucrose; cyan, DA with sucrose; green, DN without sucrose; magenta, DA without sucrose. For details, see the legend to Fig. 1. Counts of streptococci were significantly (*P* < 0.001) higher in cariogenic plaques dosed with DN and with DA. Resting plaques dosed with DN had significantly (*P* < 0.001) higher counts of streptococci than those dosed with DA.

**FIG 4:**
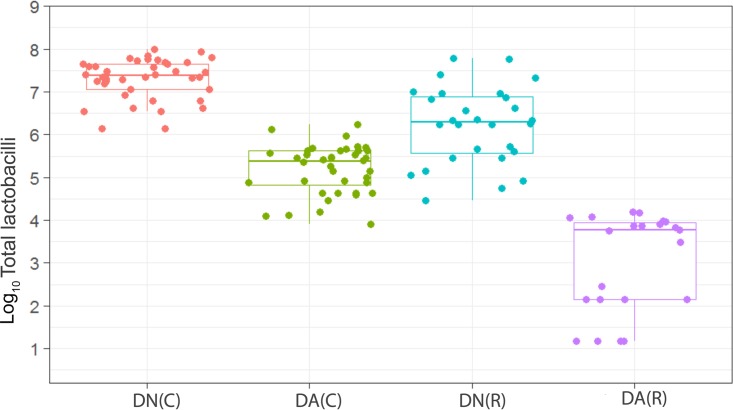
Distribution of the viable counts of lactobacilli in plaques dosed with (DA) and without (DN) arginine dentifrice in the presence (C) and absence (R) of sucrose. Box plots: red, DN with sucrose; cyan, DA with sucrose; green, DN without sucrose; magenta, DA without sucrose. For details, see the legend to Fig. 1. DN plaque counts in the presence of sucrose were significantly (*P* < 0.001) higher than DN and DA plaque counts in the absence of sucrose. DN counts in the absence of sucrose were significantly (*P* < 0.001) higher than DA plaque counts in the absence of sucrose. Counts of lactobacilli were significantly (*P* < 0.001) higher in cariogenic plaques dosed with DN and with DA. Resting plaques dosed with DN had significantly (*P* < 0.001) higher counts of lactobacilli than those dosed with DA.

**FIG 5:**
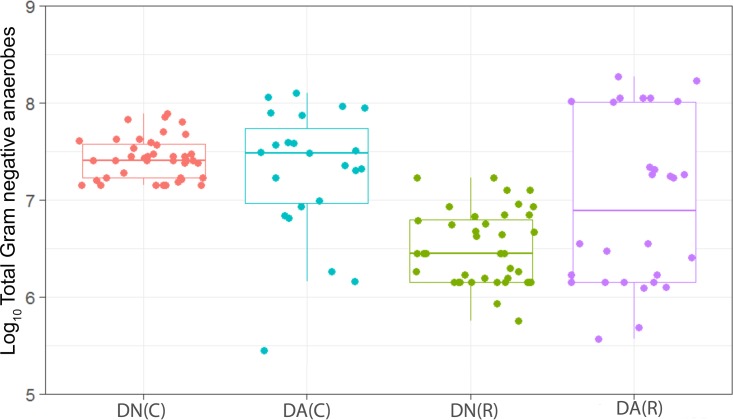
Distribution of the viable counts of Gram-negative anaerobes in plaques dosed with (DA) and without (DN) arginine dentifrice in the presence (C) and absence (R) of sucrose. Box plots: red, DN with sucrose; cyan, DA with sucrose; green, DN without sucrose; magenta, DA without sucrose. For details, see the legend to Fig. 1. Counts of Gram-negative anaerobes were significantly (*P* < 0.001) higher in cariogenic plaques dosed with DN than in resting plaques. Cariogenic and resting plaques dosed with DA had significantly higher counts than plaques dosed with DN (*P* = 0.015 and *P* = 0.004, respectively).

**FIG 6:**
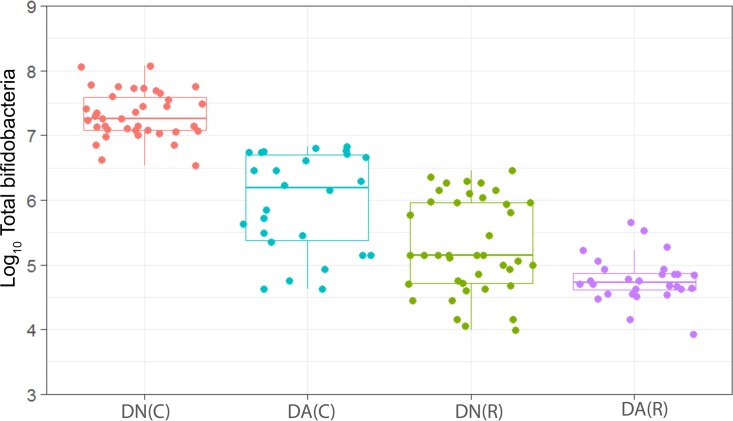
Distribution of the viable counts of bifidobacteria in plaques dosed with (DA) and without (DN) arginine dentifrice in the presence (C) and absence (R) of sucrose. Box plots: red, DN with sucrose; cyan, DA with sucrose; green, DN without sucrose; magenta, DA without sucrose. For details, see the legend to Fig. 1. Counts of bifidobacteria were significantly (*P* < 0.001) higher in cariogenic plaques dosed with DN and with DA. Resting plaques dosed with DN had significantly (*P* < 0.001) higher counts of bifidobacteria than those dosed with DA.

**FIG 7:**
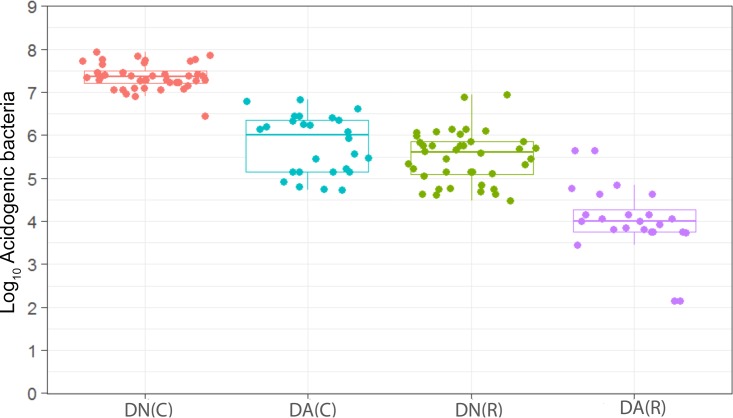
Distribution of the viable counts of acid-producing species in plaques dosed with (DA) and without (DN) arginine dentifrice in the presence (C) and absence (R) of sucrose. Box plots: red, DN with sucrose; cyan, DA with sucrose; green, DN without sucrose; magenta, DA without sucrose. For details, see the legend to Fig. 1. Counts of acidogenic bacteria were significantly (*P* < 0.001) higher in cariogenic plaques dosed with DN and with DA. Resting plaques dosed with DN had significantly (*P* < 0.001) higher counts of acidogenic bacteria than those dosed with DA.

**FIG 8:**
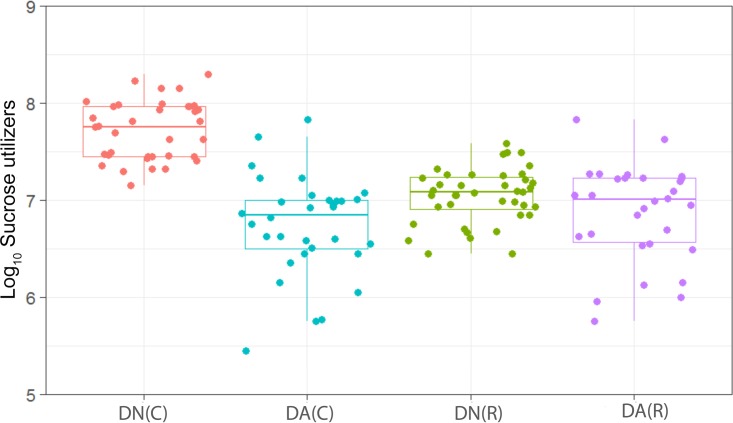
Distribution of the viable counts of sucrose-utilizing species in plaques dosed with (DA) and without (DN) arginine dentifrice in the presence (C) and absence (R) of sucrose. Box plots: red, DN with sucrose; cyan, DA with sucrose; green, DN without sucrose; magenta, DA without sucrose. For details, see the legend to Fig. 1. Counts of sucrose-utilizing bacteria were significantly (*P* < 0.001) higher in cariogenic plaques dosed with DN and with DA. Counts of sucrose-utilizing bacteria in resting plaques dosed with DN were not significantly (*P* = 0.065) changed in comparison to those dosed with DA.

**FIG 9:**
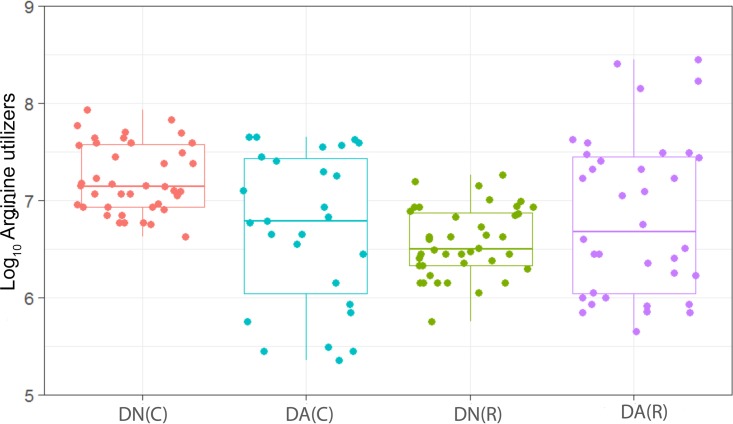
Distribution of the viable counts of arginine-utilizing species in plaques dosed with (DA) and without (DN) arginine dentifrice in the presence (C) and absence (R) of sucrose. Box plots: red, DN with sucrose; cyan, DA with sucrose; green, DN without sucrose; magenta, DA without sucrose. For details, see the legend to Fig. 1. Counts of arginine-utilizing bacteria were significantly (*P* < 0.001) higher in cariogenic plaques dosed with DN and with DA. Counts of arginine-utilizing bacteria in resting plaques dosed with DN were not significantly (*P* = 0.065) changed in comparison to those dosed with DA.

We have observed a high level of reproducibility (for example, the total facultative anaerobes were recovered at ca. 7.5 log_10_ CFU/mm^2^ throughout) between experimental runs in the CDFF in terms of the numbers of functional groups of bacteria and, importantly, a high degree of congruence between experimental runs over 7 years, as indicated by *post hoc* analysis. Previous investigations using identical model systems have recovered, for example, total anaerobes at comparable amounts (ca. 7.5 log_10_ CFU/mm^2^) (36, 42). In the present study, CDFF-grown plaques under dynamic steady states were dosed with arginine dentifrice twice daily. Bacterial communities were closely monitored using selective culture over the experimental period (Fig. 1 to 9). The media for differential bacterial counts employed in this study were selected to enumerate bacteria that have previously been implicated in dental caries (47) (i.e., total lactobacilli and total streptococci) and to monitor functionally determined aciduric and acidogenic bacteria. Additionally, to better understand the adaption of extant plaques to markedly increased arginine concentrations, bacteria capable of utilizing arginine were also selectively enumerated. During each experimental run, the sucrose-fed fermentor supported a highly significantly (*P* < 0.001) higher population density for all of the functional bacterial groups monitored, as evidenced by increased viable counts, regardless of dentifrice addition (Fig. 1 to 9), presumably due to the relief of carbon limitation within these plaques (48). Of all of the bacterial groups monitored, this increase was most marked among the lactobacilli, bifidobacteria, streptococci, and total aciduric species within fermentors supplemented with sucrose. For example, the lactobacilli (Fig. 4) accounted for ca. 10% and 0.01% of the total anaerobic counts for sucrose-supplemented and non-sucrose-supplemented fermentors, respectively. Enrichment of this important saccharolytic and acid-tolerant group of bacteria is likely to render a plaque more cariogenic, in line with the ecological plaque hypothesis (2). The numbers of all members of the monitored functional groups of bacteria were significantly (*P* < 0.05) decreased throughout a sucrose challenge during exposure to arginine in formulation (dentifrice with arginine added [DA]). This supports the daily use of dentifrice as an oral hygiene measure regardless of mechanical removal of plaque (49). This finding is in agreement with He et al. (50), who found that exposure to 1.5% arginine substantially reduced the amounts of insoluble exopolysaccharides (EPSs) in an oral biofilm. Interestingly, DA caused only a significant decrease in cariogenic and arginolytic groups of bacteria when no sucrose was present. This suggests that the plaque buffering potential and therefore anticaries effects of DA are significant, regardless of a sucrose challenge. Dentifrice without arginine added (DN) did not significantly reduce any bacterial groups in comparison to DA, except the total anaerobes and Gram-negative anaerobes, which were both significantly (*P* < 0.005) increased during DN dosing, but only in resting plaques. This suggests that the effects of arginine are more significant in cariogenic plaques.

### Effect of arginine on plaque pH.

**FIG 10:**
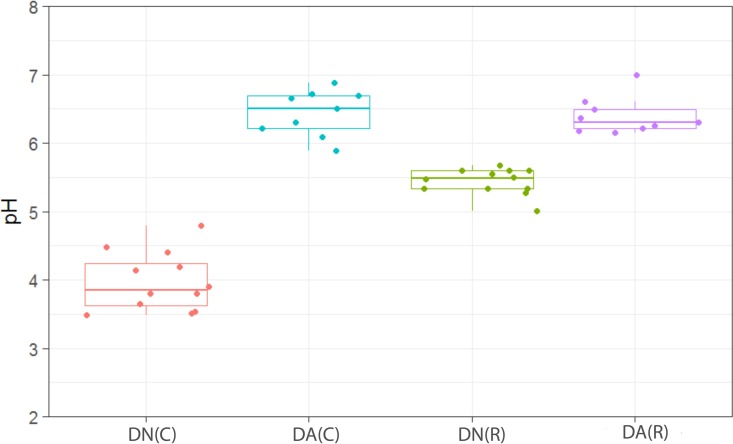
Distribution of pH measurements in plaques dosed with (DA) and without (DN) arginine dentifrice in the presence (C) and absence (R) of sucrose. Box plots: red, DN with sucrose; cyan, DA with sucrose; green, DN without sucrose; magenta, DA without sucrose. For details, see the legend to Fig. 1. pH was significantly (*P* < 0.001) higher in plaques during dosing with DA than in those dosed with DN in both resting and cariogenic plaques.

Dosing with arginine dentifrice was associated with highly significant (*P* < 0.001) increases in plaque pH (Fig. 10), which is notable in terms of previous human volunteer studies demonstrating a significant anticaries effect. In the fermentor dosed with DA (Fig. 10), the pH increase associated with dosing was gradual, taking ca. 10 days to reach maximum levels, and pH only decreased by 0.5 U after 7 days of cessation of dosing (data not shown). The pH of the plaques in the DN-dosed fermentor did not significantly increase during exposure, supporting the conclusion that arginine significantly increases plaque pH by increasing bacterial production of alkaline metabolic products.

### Analyses of bacteriological composition by DNA profiling.

**FIG 11:**
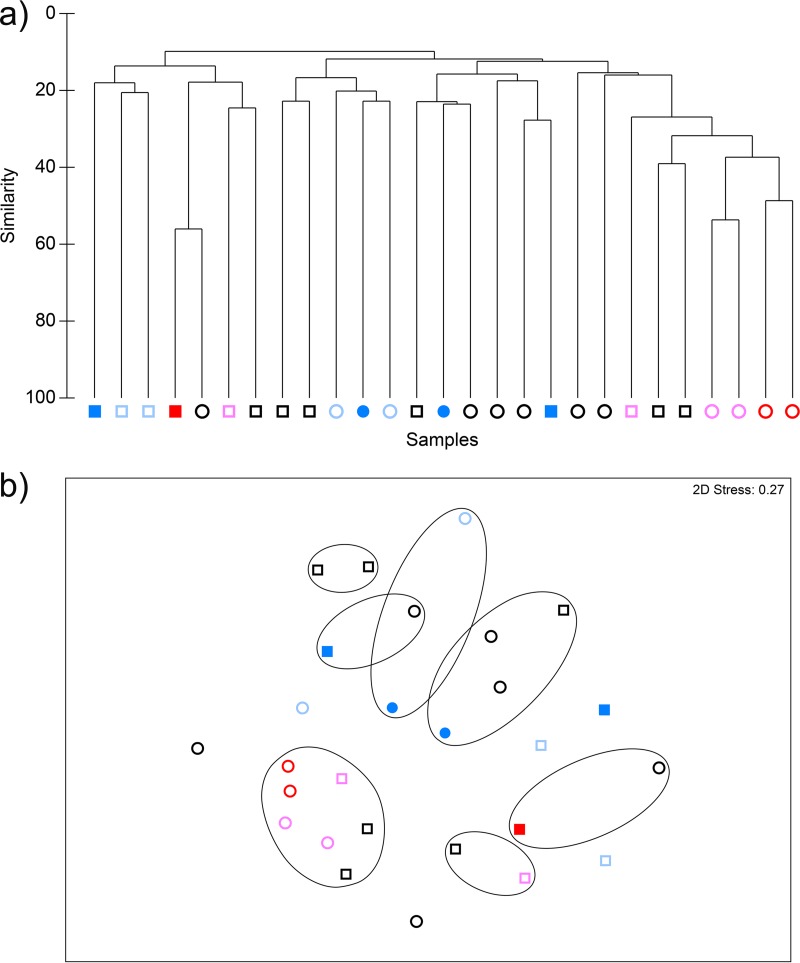
CDFF plaques analyzed by cluster analysis (a) and nonmetric MDS (b) in fermentors simulating cariogenic (square symbols) or resting (round symbols) plaques through the addition of sucrose (5% [wt/vol]) or water (dosed every 6 h), respectively. Dark and light blue symbols, respectively, indicate plaques during and after exposure to arginine dentifrice. Red and pink symbols, respectively, indicate plaques during and after exposure to nonarginine dentifrice. Black symbols indicate fermentors before dentifrice addition. Contour lines on the MDS plot superimpose 20% resemblance levels derived from the cluster analysis. Samples grouped significantly only based on sucrose addition (*P* < 0.006). Samples did not cluster significantly within runs based on exposure to arginine.

PCR-DGGE of CDFF plaques before, during, and after dosing was combined with multidimensional scaling analyses (MDS) (Fig. 11). Data thus generated provide information regarding the effects of arginine dosing on the eubacteria that may not necessarily be detected by using culture alone. According to these analyses, samples grouped significantly only on the basis of fermentor run (*P* < 0.001) and sucrose addition (*P* < 0.006). No significant groupings were observed that related to the addition of either DN or DA.

### Conclusion.

Exposure of dental plaque microcosms to arginine in dentifrice formulations significantly decreased plaque acidification and cariogenic species.

## MATERIALS AND METHODS

### Maintenance of oral microcosms.

Oral microcosms were maintained in constant-depth film fermentors (CDFFs) as previously described (36, 39, 42, 51, 52). The temperature (36°C) was maintained by locating the fermentors within Perspex incubation chambers (Stuart Scientific, Redhill, Surrey, United Kingdom). The CDFF plugs were set to a depth of 200 μm, and the rotor speed was 3 rpm. A modified artificial saliva medium was used (44, 53) that contains the following (grams per liter in distilled water): 2.5 g/liter mucin (type II, porcine, gastric), 2.0 g/liter bacteriological peptone, 2.0 g/liter tryptone, 1.0 g/liter yeast extract, 0.35 g/liter NaCl, 0.2 g/liter KCl, 0.2 g/liter CaCl_2_, 0.1 g/liter cysteine hydrochloride, 0.001 g/liter hemin, and 0.0002 g/liter vitamin K_1_. Sucrose (5% [wt/vol]) or sterile water was added (8 ml/h) to the appropriate fermentor intermittently (every 6 h) for 10 min. Saliva used for inoculation was obtained from one healthy adult female, (32 years old). Prior to inoculation, the polytetrafluoroethylene (PTFE) plug surfaces were conditioned for 24 h with artificial saliva, which was continuously added at 8 ml/h to each fermentor by a peristaltic pump (Minipuls 3; Gilson). Hydroxyapatite was not used as substrata since, due to long-term growth of acidogenic plaques, dissolution may have occurred at different rates in different fermentors, thus representing an additional experimental variable.

The fermentors were inoculated with fresh saliva on three separate occasions (2.0 ± 0.5 ml/fermentor/inoculation) over a period of 24 h using fresh, pooled saliva from the donor. Once dynamic steady states were established (which took approximately 7 days, as evidenced by stability of the colony counts and plaque pH analysis), arginine dentifrice (10% slurry in sterile distilled water) dosing began at a regimen of every 6 h for 10 min (8 ml/h) immediately following the sucrose pulse. The dentifrice slurries were continuously mixed. Dosing continued for up to 3 weeks, after which fermentors were maintained without arginine or dentifrice addition for an additional 10 days. Samples were taken aseptically at regular intervals throughout the experimental periods. Sampled CDFF pans were immediately aseptically replaced with sterile pans. Pans were numbered and sampled sequentially in order to avoid analyzing immature plaques. Samples were processed in less than 30 min for bacteriology and pH analysis or were archived at −60°C for subsequent analysis by PCR-DGGE.

### Dentifrices.

The formulations tested comprised an arginine formulation containing 1.5% (wt/wt) arginine bicarbonate, 1,450 ppm sodium monofluorophosphate (DA), and 10% calcium carbonate and a control toothpaste (as described above but without arginine [DN]). Both formulations were obtained from Colgate-Palmolive (Piscataway, NJ).

### Differential bacteriological analysis.

For enumeration, samples of microcosm plaques (two sample plugs) were homogenized by vortex mixing for 1 min. The samples were then serially diluted with prereduced, half-strength thioglycolate medium (USP). Appropriate dilutions (0.1 ml) were then plated in triplicate onto a variety of selective and nonselective media. These media were Wilkins-Chalgren (WC) agar for total anaerobes and total facultative anaerobes, WC agar with supplements for Gram-negative organisms for total Gram-negative anaerobes, WC agar reduced to pH 4.5 using lactic acid for total aciduric bacteria (54), WC agar with 5% (wt/vol) sucrose, 5% (wt/vol) glucose, and 0.05% (wt/vol) bromocresol purple for total acidogenic bacteria (adapted from reference 55), Rogosa agar for total lactobacilli, Trypticase yeast extract, cysteine, and sucrose agar for *Streptococcus* spp., Beerens agar (54) for total bifidobacteria, and decarboxylase agar with 10% arginine (wt/vol) containing (in grams per liter in distilled water) 5.0 g/liter bacteriological peptone, 3.0 g/liter yeast extract, 1.0 g/liter dextrose, and 0.05 g/liter bromocresol purple for total arginine-utilizing bacteria (modified from reference 56). Positive acidogenic and arginine-utilizing bacteria are indicated by the appearance of yellow or purple colonies, respectively. These agars were immediately placed in an anaerobic chamber (Don Whitley Scientific, Shipley, United Kingdom), with an atmosphere of 10% H_2_, 10% CO_2_, and 80% N_2_. All agars except WC plates for total facultative anaerobes were maintained at 37°C for up to 5 days; the facultative anaerobic plates were incubated aerobically at 37°C in a standard incubator for 3 days. After this time, appropriate dilutions were counted.

### pH analysis of microcosm plaques.

After 2 plugs were removed from a CDFF pan for bacteriological analysis, a needle pH electrode (MI-407; Microelectrodes, Inc., Bedford, NH) was inserted to a depth of approximately 200 µm into the middle of one of the remaining plaques, and the pH was recorded. The electrode was coupled to a Jenway 3510 pH meter (Bibby Scientific, Ltd., Staffordshire, United Kingdom) and was calibrated prior to analyses.

### Bacteriological analysis by PCR-DGGE.

DNA was extracted from the archived CDFF samples using a DNA stool minikit (Qiagen, West Sussex, United Kingdom) in accordance with the manufacturer’s instructions and analyzed by PCR-DGGE as previously described (51).

### Dendrogram construction for cluster analysis.

Gel images were aligned using Adobe Photoshop Elements (Adobe Systems, San Jose, CA). Gel images were then analyzed using the BioNumerics software package (version 4.5, Applied Maths, Sint-Martens, Belgium). The bands present in each lane were detected automatically and then checked manually. A reference lane was created using bands present in each lane, generate matching profiles. The matching profiles for each lane were used to produce a dendrogram by the unweighted pair group method with arithmetic mean (UPGMA [57]) so that clustering patterns could be determined.

### Analysis of DNA profiles.

DNA profiles were analyzed as described by Kampoo et al. (55). Briefly, negative images of stained DGGE gels were aligned using Adobe Photoshop CS6 (Adobe, London, United Kingdom) and then analyzed with BioNumerics v.5.1 (Applied Maths, Sint-Martens-Latem, Belgium). Lanes on gel images were selected manually and then compared to reference lanes. To test for potential differences in CDFF plaque composition, binary band-matching profiles for each lane were analyzed with PRIMER software (v.6) (Primer-E, Ltd., Luton, United Kingdom) as follows. Bray-Curtis similarity values were calculated for imported binary gel band data, and agglomerative hierarchical clustering was done via the CLUSTER menu of the PRIMER software. Similarity profile permutation tests were used to test for statistically significant evidence of genuine clusters, and data were further analyzed by using the nonmetric multidimensional scaling (MDS) algorithm. To test the significance of potential differences in bacteriological profiles, analysis of similarity (ANOSIM) was done with the ANOSIM test.

### Statistical modeling.

Bacterial count and pH were analyzed and plotted using the free software R. Although there is a time component within the experiments (when dentifrice dosing commences and ceases), the same timings and duration were used for all experiments. Thus, the statistical evaluation can be simplified by analyzing the distribution of viable counts or pH measurements for a given variable between treatments of interest over the first 14 days. This was first explored graphically via box plots before analyzing the average variable count across the whole time series using linear regression. The variables of interest that were explored were (i) average variable counts with and without a sucrose challenge, (ii) average variable counts with a sucrose challenge with and without DA dosing, and (iii) average variable counts without a sucrose challenge with and without DA dosing. *P* values from the *F* test were reported as significant changes if the *P* value was <0.05.

## References

[1] Bagramian RA, Garcia-Godoy F, Volpe AR 2009 The global increase in dental caries. A pending public health crisis. Am J Dent 22:3–8. 19281105

[2] Marsh PD 1994 Microbial ecology of dental plaque and its significance in health and disease. Adv Dent Res 8:263–271. doi:10.1177/08959374940080022001. 7865085

[3] Marsh PD 2003 Plaque as a biofilm: pharmacological principles of drug delivery and action in the sub- and supragingival environment. Oral Dis 9:16–22. doi:10.1034/j.1601-0825.9.s1.4.x. 12974526

[4] Marsh PD 1991 Dentifrices containing new agents for the control of plaque and gingivitis: microbiological aspects. J Clin Periodontol 18:462–467. doi:10.1111/j.1600-051X.1991.tb02317.x. 1890229

[5] Kleinberg I 1967 Effect of urea concentration on human plaque pH levels in situ. Arch Oral Biol 12:1475–1484. doi:10.1016/0003-9969(67)90183-5. 5237332

[6] Kleinberg I 2002 SensiStat. A new saliva-based composition for simple and effective treatment of dentinal sensitivity pain. Dent Today 21:42–47. 12572161

[7] Petrou I, Heu R, Stranick M, Lavender S, Zaidel L, Cummins D, Sullivan RJ, Hsueh C, Gimzewski JK 2009 A breakthrough therapy for dentin hypersensitivity: how dental products containing 8% arginine and calcium carbonate work to deliver effective relief of sensitive teeth. J Clin Dent 20:23–31. 19489189

[8] Cummins D 2009 Dentin hypersensitivity: from diagnosis to a breakthrough therapy for everyday sensitivity relief. J Clin Dent 20:1–9. 19489186

[9] García-Godoy F 2009 Dentin hypersensitivity: beneficial effects of an arginine-calcium carbonate desensitizing paste. Am J Dent 22:2A. 19472554

[10] Panagakos FS, Volpe AR, Petrone ME, DeVizio W, Davies RM, Proskin HM 2005 Advanced oral antibacterial/anti-inflammatory technology: a comprehensive review of the clinical benefits of a triclosan/copolymer/fluoride dentifrice. J Clin Dent 16:S1–S19. 16583598

[11] Panagakos F, Schiff T, Guignon A 2009 Dentin hypersensitivity: effective treatment with an in-office desensitizing paste containing 8% arginine and calcium carbonate. Am J Dent 22:3A–7A. 19472555

[12] Hamlin D, Williams KP, Delgado E, Zhang YP, DeVizio W, Mateo LR 2009 Clinical evaluation of the efficacy of a desensitizing paste containing 8% arginine and calcium carbonate for the in-office relief of dentin hypersensitivity associated with dental prophylaxis. Am J Dent 22:16A–20A. 19472557

[13] Schiff T, Delgado E, Zhang YP, Cummins D, DeVizio W, Mateo LR 2009 Clinical evaluation of the efficacy of an in-office desensitizing paste containing 8% arginine and calcium carbonate in providing instant and lasting relief of dentin hypersensitivity. Am J Dent 22:8A–15A. 19472556

[14] Ayad F, Ayad N, Zhang YP, DeVizio W, Cummins D, Mateo LR 2009 Comparing the efficacy in reducing dentin hypersensitivity of a new toothpaste containing 8.0% arginine, calcium carbonate, and 1450 ppm fluoride to a commercial sensitive toothpaste containing 2% potassium ion: an eight-week clinical study on Canadian adults. J Clin Dent 20:10–16. 19489187

[15] Docimo R, Montesani L, Maturo P, Costacurta M, Bartolino M, DeVizio W, Zhang YP, Cummins D, Dibart S, Mateo LR 2009 Comparing the efficacy in reducing dentin hypersensitivity of a new toothpaste containing 8.0% arginine, calcium carbonate, and 1450 ppm fluoride to a commercial sensitive toothpaste containing 2% potassium ion: an eight-week clinical study in Rome, Italy. J Clin Dent 20:17–22. 19489188

[16] Kraivaphan P, Amornchat C, Triratana T, Mateo LR, Ellwood R, Cummins D, DeVizio W, Zhang YP 2013 Two-year caries clinical study of the efficacy of novel dentifrices containing 1.5% arginine, an insoluble calcium compound and 1,450 ppm fluoride. Caries Res 47:582–590. doi:10.1159/000353183. 23988908

[17] Li X, Zhong Y, Jiang X, Hu Deyu D, Mateo LR, Morrison BM Jr, Zhang YP 2015 Randomized clinical trial of the efficacy of dentifrices containing 1.5% arginine, an insoluble calcium compound and 1450 ppm fluoride over two years. J Clin Dent 26:7–12. 26054185

[18] Hu DY, Yin W, Li X, Feng Y, Zhang YP, Cummins D, Mateo LR, Ellwood RP 2013 A clinical investigation of the efficacy of a dentifrice containing 1.5% arginine and 1450 ppm fluoride, as sodium monofluorophosphate in a calcium base, on primary root caries. J Clin Dent 24:A23–A31. 24156137

[19] Acevedo AM, Montero M, Rojas-Sanchez F, Machado C, Rivera LE, Wolff M, Kleinberg I 2008 Clinical evaluation of the ability of CaviStat in a mint confection to inhibit the development of dental caries in children. J Clin Dent 19:1–8. 18500152

[20] Acevedo AM, Machado C, Rivera LE, Wolff M, Kleinberg I 2005 The inhibitory effect of an arginine bicarbonate/calcium carbonate CaviStat-containing dentifrice on the development of dental caries in Venezuelan school children. J Clin Dent 16:63–70. 16305004

[21] Vranić L, Granić P, Rajić Z 1991 Basic amino acid in the pathogenesis of caries. Acta Stomatol Croat 25:71–76. 1819935

[22] Perinpanayagam HE, Van Wuyckhuyse BC, Ji ZS, Tabak LA 1995 Characterization of low-molecular-weight peptides in human parotid saliva. J Dent Res 74:345–350. doi:10.1177/00220345950740011001. 7876428

[23] Burne RA, Marquis RE 2000 Alkali production by oral bacteria and protection against dental caries. FEMS Microbiol Lett 193:1–6. doi:10.1111/j.1574-6968.2000.tb09393.x. 11094270

[24] Higham SM, Edgar WM 1989 Human dental plaque pH, and the organic acid and free amino acid profiles in plaque fluid, after sucrose rinsing. Arch Oral Biol 34:329–334. doi:10.1016/0003-9969(89)90105-2. 2597027

[25] Nascimento MM, Gordan VV, Garvan CW, Browngardt CM, Burne RA 2009 Correlations of oral bacterial arginine and urea catabolism with caries experience. Oral Microbiol Immunol 24:89–95. doi:10.1111/j.1399-302X.2008.00477.x. 19239634 PMC2742966

[26] Huang X, Exterkate RA, ten Cate JM 2012 Factors associated with alkali production from arginine in dental biofilms. J Dent Res 91:1130–1134. doi:10.1177/0022034512461652. 23010718

[27] ten Cate JM, Cummins D 2013 Fluoride toothpaste containing 1.5% arginine and insoluble calcium as a new standard of care in caries prevention. J Clin Dent 24:79–87. 24660269

[28] Marquis RE, Bender GR, Murray DR, Wong A 1987 Arginine deiminase system and bacterial adaptation to acid environments. Appl Environ Microbiol 53:198–200. 3103530 10.1128/aem.53.1.198-200.1987 PMC203628

[29] Rogers AH 1990 Utilization of nitrogenous compounds by oral bacteria. Aust Dent J 35:468–471. doi:10.1111/j.1834-7819.1990.tb05432.x. 2073196

[30] Socransky SS, Haffajee AD, Cugini MA, Smith C, Kent RL Jr 1998 Microbial complexes in subgingival plaque. J Clin Periodontol 25:134–144. doi:10.1111/j.1600-051X.1998.tb02419.x. 9495612

[31] Jakubovics NS, Robinson JC, Samarian DS, Kolderman E, Yassin SA, Bettampadi D, Bashton M, Rickard AH 2015 Critical roles of arginine in growth and biofilm development by *Streptococcus gordonii*. Mol Microbiol 97:281–300. doi:10.1111/mmi.13023. 25855127

[32] Nascimento MM, Liu Y, Kalra R, Perry S, Adewumi A, Xu X, Primosch RE, Burne RA 2013 Oral arginine metabolism may decrease the risk for dental caries in children. J Dent Res 92:604–608. doi:10.1177/0022034513487907. 23640952 PMC3684231

[33] Cummins D 2009 The efficacy of a new dentifrice containing 8.0% arginine, calcium carbonate, and 1450 ppm fluoride in delivering instant and lasting relief of dentin hypersensitivity. J Clin Dent 20:109–114. 19831163

[34] Kolderman E, Bettampadi D, Samarian D, Dowd SE, Foxman B, Jakubovics NS, Rickard AH 2015 l-Arginine destabilizes oral multi-species biofilm communities developed in human saliva. PLoS One 10:e0121835. doi:10.1371/journal.pone.0121835. 25946040 PMC4422691

[35] Koopman JE, Hoogenkamp MA, Buijs MJ, Brandt BW, Keijser BJ, Crielaard W, ten Cate JM, Zaura E 2017 Changes in the oral ecosystem induced by the use of 8% arginine toothpaste. Arch Oral Biol 73:79–87. doi:10.1016/j.archoralbio.2016.09.008. 27697693

[36] McBain AJ, Bartolo RG, Catrenich CE, Charbonneau D, Ledder RG, Gilbert P 2003 Effects of triclosan-containing rinse on the dynamics and antimicrobial susceptibility of in vitro plaque ecosystems. Antimicrob Agents Chemother 47:3531–3538. doi:10.1128/AAC.47.11.3531-3538.2003. 14576113 PMC253811

[37] Shapiro S, Giertsen E, Guggenheim B 2002 An in vitro oral biofilm model for comparing the efficacy of antimicrobial mouthrinses. Caries Res 36:93–100. 12037365 10.1159/000057866

[38] Finney M, Walker JT, Marsh PD, Brading MG 2003 Antimicrobial effects of a novel triclosan/zinc citrate dentifrice against mixed culture oral biofilms. Int Dent J 53:371–378. doi:10.1111/j.1875-595X.2003.tb00912.x. 14725381

[39] McBain AJ, Bartolo RG, Catrenich CE, Charbonneau D, Ledder RG, Gilbert P 2003 Effects of a chlorhexidine gluconate-containing mouthwash on the vitality and antimicrobial susceptibility of in vitro oral bacterial ecosystems. Appl Environ Microbiol 69:4770–4776. doi:10.1128/AEM.69.8.4770-4776.2003. 12902270 PMC169085

[40] Kinniment SL, Wimpenny JW, Adams D, Marsh PD 1996 The effect of chlorhexidine on defined, mixed culture oral biofilms grown in a novel model system. J Appl Bacteriol 81:120–125. doi:10.1111/j.1365-2672.1996.tb04488.x. 8760321

[41] Pratten J, Barnett P, Wilson M 1998 Composition and susceptibility to chlorhexidine of multispecies biofilms of oral bacteria. Appl Environ Microbiol 64:3515–3519. 9726908 10.1128/aem.64.9.3515-3519.1998 PMC106758

[42] Ledder RG, Madhwani T, Sreenivasan PK, De Vizio W, McBain AJ 2009 An *in vitro* evaluation of hydrolytic enzymes as dental plaque control agents. J Med Microbiol 58:482–491. doi:10.1099/jmm.0.006601-0. 19273645

[43] Owens GJ, Lynch RJM, Hope CK, Cooper L, Higham SM, Valappil SP 2017 Evidence of an in vitro coupled diffusion mechanism of lesion formation within microcosm dental plaque. Caries Res 51:188–197. doi:10.1159/000456015. 28245470

[44] Pratten J, Wilson M 1999 Antimicrobial susceptibility and composition of microcosm dental plaques supplemented with sucrose. Antimicrob Agents Chemother 43:1595–1599. 10390209 10.1128/aac.43.7.1595 PMC89330

[45] Guggenheim B, Giertsen E, Schüpbach P, Shapiro S 2001 Validation of an *in vitro* biofilm model of supragingival plaque. J Dent Res 80:363–370. doi:10.1177/00220345010800011201. 11269730

[46] Hope CK, Bakht K, Burnside G, Martin GC, Burnett G, de Josselin de Jong E, Higham SM 2012 Reducing the variability between constant-depth film fermenter experiments when modelling oral biofilm. J Appl Microbiol 113:601–608. doi:10.1111/j.1365-2672.2012.05368.x. 22716966

[47] Takahashi N, Nyvad B 2008 Caries ecology revisited: microbial dynamics and the caries process. Caries Res 42:409–418. doi:10.1159/000159604. 18832827

[48] Guggenheim B 1970 Extracellular polysaccharides and microbial plaque. Int Dent J 20:657–678. 5276615

[49] Xu T, Herles SM, Barnes VM 2004 New laboratory methods to study tooth surface coverage and interproximal plaque control by dentifrice products. J Clin Dent 15:123–127. 15794458

[50] He J, Hwang G, Liu Y, Gao L, Kilpatrick-Liverman L, Santarpia P, Zhou X, Koo H 2016 l-Arginine modifies the exopolysaccharide matrix and thwarts *Streptococcus mutans* outgrowth within mixed-species oral biofilms. J Bacteriol 198:2651–2661. doi:10.1128/JB.00021-16. 27161116 PMC5019072

[51] McBain AJ, Bartolo RG, Catrenich CE, Charbonneau D, Ledder RG, Gilbert P 2003 Growth and molecular characterization of dental plaque microcosms. J Appl Microbiol 94:655–664. doi:10.1046/j.1365-2672.2003.01876.x. 12631201

[52] Wilson M 1999 Use of constant depth film fermentor in studies of biofilms of oral bacteria. Methods Enzymol 310:264–279. 10547799 10.1016/s0076-6879(99)10023-5

[53] Shah HN, Williams RA, Bowden GH, Hardie JM 1976 Comparison of the biochemical properties of *Bacteroides melaninogenicus* from human dental plaque and other sites. J Appl Bacteriol 41:473–495. doi:10.1111/j.1365-2672.1976.tb00660.x. 14095

[54] Beerens H 1990 An elective and selective isolation medium for *Bifidobacterium* spp. Lett Appl Microbiol 11:155–157. doi:10.1111/j.1472-765X.1990.tb00148.x.

[55] Kampoo K, Teanpaisan R, Ledder RG, McBain AJ 2014 Oral bacterial communities in individuals with type 2 diabetes who live in southern Thailand. Appl Environ Microbiol 80:662–671. doi:10.1128/AEM.02821-13. 24242241 PMC3911101

[56] Jones RN, Fuchs PC, Sniderman S 1976 Comparison of amino acid decarboxylase and dehydrolase results by Moeller, rapid, and replicator plate methods. J Clin Microbiol 3:75–76. 767360 10.1128/jcm.3.1.75-76.1976 PMC274231

[57] Sneath PH, Sokal RR 1973 Numerical taxonomy. Freeman, London, United Kingdom.

